# Cadonilimab combined with trastuzumab and chemotherapy for HER2-positive gastric cancer with bone marrow metastasis and DIC: a case report and literature review

**DOI:** 10.3389/fimmu.2025.1608636

**Published:** 2025-06-30

**Authors:** Meijing Chen, Jiwen Fan, Danyang Zhou, Boran Cheng, Shubin Wang, Gangling Tong

**Affiliations:** Department of Oncology, Peking University Shenzhen Hospital, Affiliated Hospital of Guangdong Medical University, Shenzhen Key Laboratory of Gastrointestinal Cancer Translational Research, Cancer Institute of Shenzhen-Peking University-Hong Kong University of Science and Technology (PKU-HKUST) Medical Center, Shenzhen, Guangdong, China

**Keywords:** gastric cancer (GC), bone marrow metastasis (BMM), disseminated intravascular coagulation (DIC), HER-2, PD-L1, immunotherapy, chemotherapy

## Abstract

**Background:**

Gastric cancer (GC) is one of the most common malignancies worldwide. While bone marrow metastasis (BMM) in GC is extremely rare and often complicated by disseminated intravascular coagulation (DIC), a critical condition with a median survival of less than three months in untreated patients. Human epidermal growth factor receptor-2 (HER-2) plays a critical role in GC pathogenesis, and trastuzumab-based regimens have significantly improved outcomes in HER-2 positive metastatic GC. However, the efficacy of immune checkpoint inhibitors (ICIs) in HER-2 positive GC patients with BMM and DIC, particularly those with programmed death-ligand 1 (PD-L1) combined positive score (CPS) <1, remains unclear. Here, we present a case of GC with BMM and DIC that achieved long-term survival through treatment with cadonilimab (an anti-PD-1/CTLA-4 bispecific antibody) in combination with trastuzumab and chemotherapy. Through this case and literature review, we aim to explore optimal treatment strategies for this rare and challenging subgroup.

**Case presentation:**

A 35-year-old woman presented with left hip pain. Imaging and lab tests indicated bone metastasis and DIC. Further evaluation with PET/CT, gastroscopy, and biopsy confirmed poorly differentiated gastric adenocarcinoma with bone marrow involvement (cT3N+M1, stage IVB). Immunohistochemistry demonstrated PD-L1 CPS <1 and HER-2 (2+), though fluorescence *in situ* hybridization (FISH) was negative. Notably, next-generation sequencing (NGS) detected a high plasma HER-2 copy number (34.88). Given her ECOG performance status of 2, initial therapy consisted of trastuzumab combined with docetaxel and fluorouracil, alongside supportive care. Within two weeks, DIC resolved, and pain significantly improved. Treatment was then escalated to a combination of cadonilimab, trastuzumab, and FLOT (5-FU, leucovorin, oxaliplatin, docetaxel). After achieving a partial response, she developed an oxaliplatin allergy, prompting a switch to maintenance therapy with cadonilimab, trastuzumab, and S-1. She achieved progression-free survival (PFS) of nearly 12 months and overall survival (OS) of approximately 15 months, with sustained quality of life throughout treatment course.

**Conclusion:**

This case demonstrates that intensive anti-tumor therapy combining HER-2-targeted agents, ICIs, and chemotherapy, alongside supportive care, can prolong survival and improve life quality in GC patients with BMM and DIC. Hematologic toxicities were the main adverse events but were tolerable, supporting the regimen’s feasibility and safety.

## 1 Introduction

Gastric cancer (GC) is the fifth most common malignant tumor worldwide, with approximately 1.08 million new cases and 760,000 deaths annually, posing a significant threat to human health (1). The clinical symptoms of early GC are nonspecific, leading to most patients being diagnosed with locally advanced disease at initial presentation (2). Despite advances in comprehensive treatments, including surgery, pharmacotherapy, and radiotherapy, which have improved the prognosis of patients with locally advanced GC, the outlook for those with metastatic GC remains poor, with a 5-year survival rate of less than 5% (3). The liver, lungs, and peritoneum are typically metastatic sites of GC, whereas bone marrow metastasis (BMM) is rare (4). BMM is a distinct subtype of bone metastasis, characterized by the dissemination of extramedullary malignant tumor cells to the bone marrow via the bloodstream. This process disrupts normal hematopoiesis, leading to a series of hematologic and clinical complications. BMM has an insidious onset, making diagnosis and treatment challenging, and is associated with a poor prognosis (5). Additionally, disseminated intravascular coagulation (DIC) is a severe coagulopathy caused by pathological activation of coagulation factors within blood vessels, often leading to organ dysfunction. DIC is a known complication of cancer and has been reported in various solid tumors, including GC (6). BMM may contribute to the development of DIC, and patients with GC complicated by DIC have a dismal prognosis, with a median overall survival (OS) of less than three months in untreated cases (7). Therefore, there is an urgent need to identify more effective treatment strategies to improve the prognosis of patients with GC complicated by BMM and DIC.

Human epidermal growth factor receptor 2 (HER-2) is a critical driver of GC progression. HER-2-positive GC represents a distinct subtype, accounting for approximately 10–20% of cases. Current diagnostic criteria define HER-2 positivity as either immunohistochemistry (IHC) 3+ or fluorescence *in situ* hybridization (FISH) positivity. While next-generation sequencing (NGS) for HER-2 copy number variation has gained FDA approval as a companion diagnostic in breast cancer, its clinical application in GC requires further validation (8). Earlier studies have demonstrated that trastuzumab-based regimens improve the prognosis of patients with HER-2-positive metastatic GC (9). The KEYNOTE-811 clinical trial revealed that the combination of pembrolizumab with trastuzumab and chemotherapy as a first-line treatment for metastatic HER-2-positive gastric or gastroesophageal junction adenocarcinoma significantly improved progression-free survival (PFS) compared to placebo, particularly in patients with PD-L1 CPS ≥1. However, in patients with PD-L1 CPS <1, the improvement in PFS was limited, and OS data are still under follow-up (10). Thus, the therapeutic benefit of ICIs in HER-2-positive, PD-L1-negative GC patients remains uncertain.

This report presents the case of a young female patient with metastatic GC complicated by BMM and DIC. Comprehensive molecular profiling revealed discordant HER-2 status: while IHC showed HER-2 (2+) with negative FISH, NGS demonstrated a remarkably high plasma HER-2 copy number (34.88). The patient initially received trastuzumab combined with chemotherapy, followed by the addition of cadonilimab (an anti-PD-1/CTLA-4 bispecific antibody) as part of the first-line treatment regimen. Notably, the patient’s OS exceeded one year, suggesting the potential efficacy of this treatment approach.

## 2 Case report

**Figure 1:**
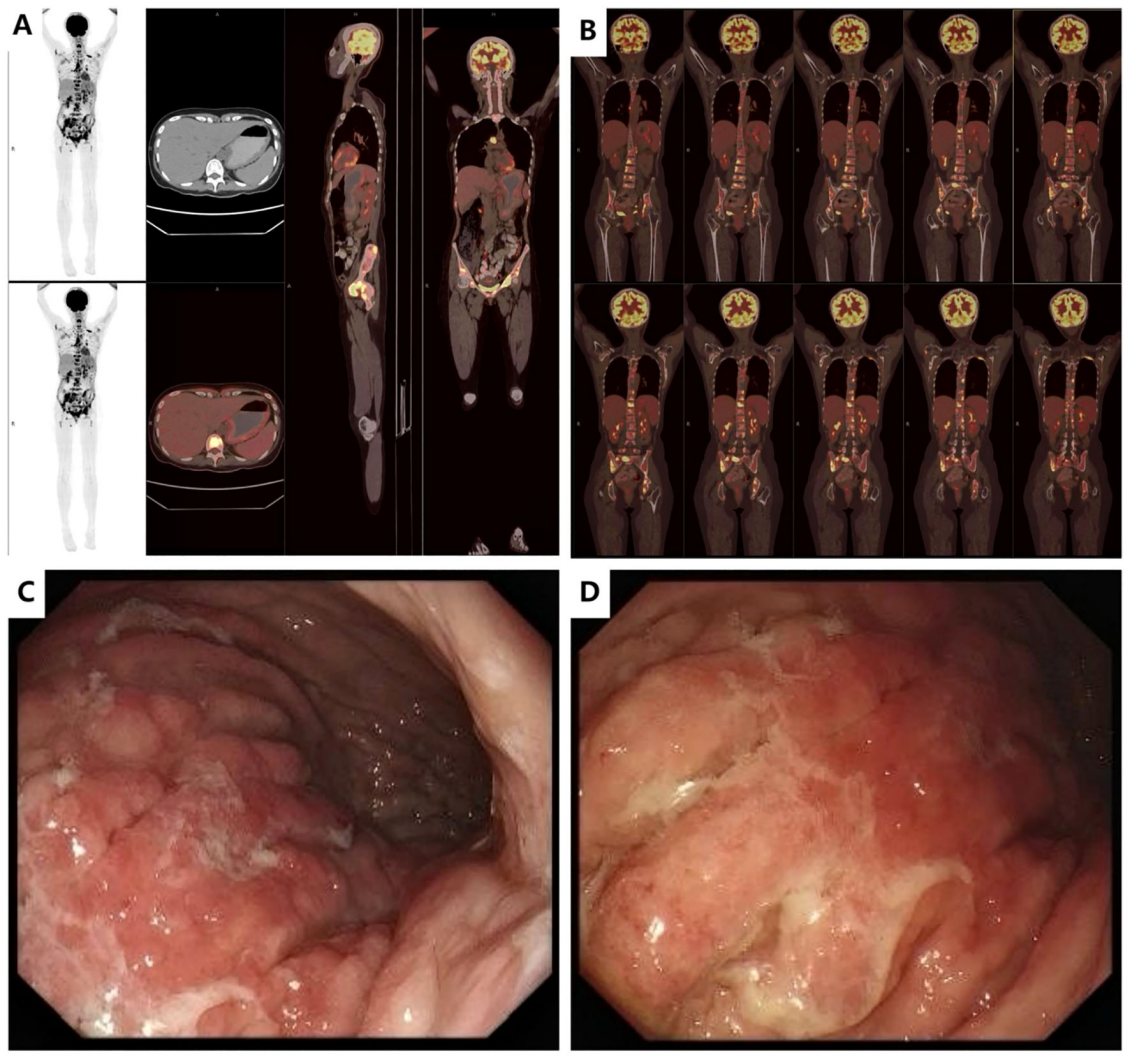
Imaging data at initial diagnosis before treatment **(A)**. PET-CT (2023-12-11): Diffusely increased metabolic activity in the gastric fundus and body with irregular wall thickening. **(B)**. PET-CT (2023-12-11): Multiple osteolytic/osteoblastic bone metastases involving the cervical, thoracic, and lumbar spine, sacrum and coccyx, sternum, bilateral scapulae, bilateral ribs, pelvic bones, and proximal femurs bilaterally. **(C, D)**. Endoscopic Ultrasound (EUS, 2023-12-11): Diffuse mucosal thickening with heterogeneous hypoechoic texture. Loss of distinct submucosal layer and partial invasion into the muscularis propria layer. Maximum wall thickness: 7.28 mm. Highly suggestive of linitis plastica (leather bottle stomach).

**Figure 2:**
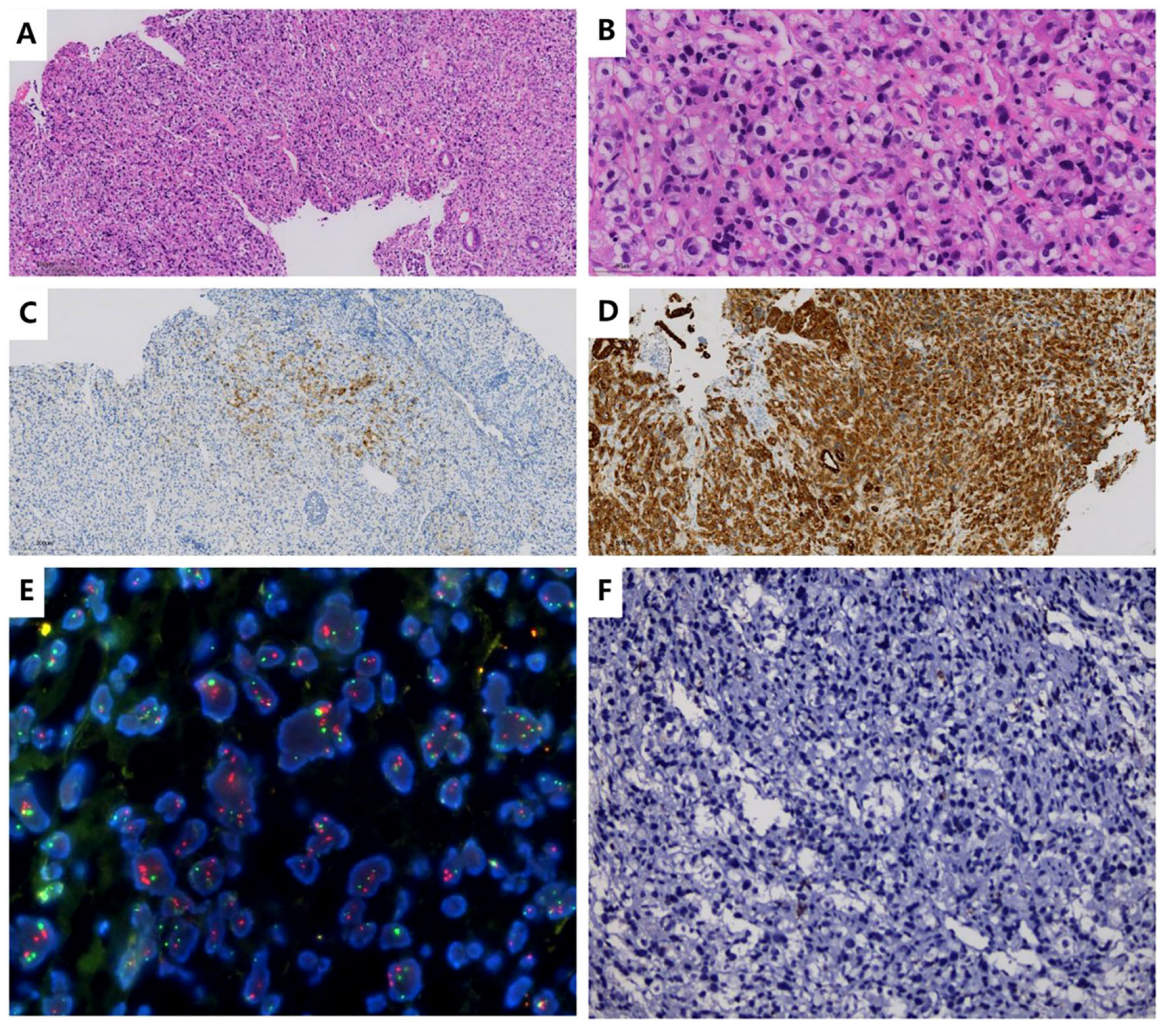
Histopathological and Immunohistochemical Findings **(A, B)**. Hematoxylin and eosin (H&E) staining: Sections show features consistent with poorly differentiated gastric adenocarcinoma. **(C)**. Immunohistochemistry (IHC) of gastric body tissue: HER-2: 2+ (equivocal by ASCO/CAP criteria). **(D)**. IHC of gastric body tissue: CK-pan: Positive (pancytokeratin expression confirmed). **(E)**. FISH of gastric body tissue: HER-2 amplification: Negative (no gene amplification detected). **(F)**. PD-L1 testing (using 22C3 antibody): Combined Positive Score (CPS): <1 (negative expression).

The patient was a 35-year-old female who was previously healthy, with no significant personal or genetic medical history. She presented in November 2023 with left hip pain, which occurred without any obvious triggers, and was rated 6 out of 10 on the Numeric Rating Scale (NRS). The pain worsened with physical activity, and the patient denied any history of trauma. A CT scan of the hip joint on December 5, 2023, revealed bone destruction in the left anterior inferior acetabulum and suprapubic branch of the acetabulum, raising concern for a neoplastic lesion. The scan also showed multiple hypodense areas in the pelvis, suggestive of possible bone destruction. Laboratory test results from December 5, 2023, were as follows: prothrombin time (PT) of 15.50 seconds, fibrinogen (FIB) level of <1.8 g/L, D-dimer >20 mg/L, and platelet count (PLT) of 50 × 10^9^/L (**Table 1**), indicating a state of DIC. The patient received 2 units of homogeneous red blood cell suspension, and her indicators were monitored regularly. On December 8, 2023, an MRI of the hip joint with contrast enhancement was performed. The results showed multiple patchy isotropic T1 and slightly prolonged T2 signal shadows with unclear boundaries in the bilateral acetabulum, pubic bone, sciatic bone, femoral head, femoral neck, proximal femoral stem, sacral vertebrae, and lumbar vertebrae. High signal intensity was observed in the fat portion, and there was enhanced contrast uptake, particularly in the left acetabulum, suggesting a metastatic tumor involving the left hip joint and surrounding soft tissues. A PET-CT scan was performed on December 11, 2023, which showed heterogeneous increases in FDG uptake in the gastric fundus, the greater curvature of the gastric body, and the posterior wall of the lesser curvature. The maximum standardized uptake value (SUVmax) ranged from 3.61 to 4.81, with an average SUV (SUVave) of 2.25 to 4.15 in early imaging, and an SUVmax of 4.14 to 5.28, with an SUVave of 3.57 to 3.52 in delayed imaging. Extensive foci of increased FDG uptake, varying in size and morphology, were observed in the cervicothoracic and lumbar vertebrae, sacrococcygeal vertebrae, sternum, bilateral scapulae, ribs, pelvic bones, and bilateral femurs. The SUVmax ranged from 4.65 to 8.20, with an SUVave of 3.86 to 4.15 in early imaging, and an SUVmax of 4.14 to 5.28, with an SUVave of 3.57 to 3.52 in delayed imaging. These findings were consistent with widespread metastatic disease (**Figures 1A, B**). Gastroscopy performed on December 12, 2023, revealed mucosal thickening, stiffness, poor peristalsis, and involvement of the greater curvature and posterior wall, with some involvement of the anterior wall, giving the stomach a leathery appearance (**Figures 1C, D**). Pathological analysis confirmed poorly differentiated gastric adenocarcinoma. Immunohistochemistry (IHC) results showed HER-2 (2+), negative FISH, and negative Epstein-Barr virus-encoded RNA (EBER). NGS revealed plasma ERBB2 gene amplification, with a copy number (CN) of 34.88, and PD-L1 CPS <1 (22C3 antibody) (**Figure 2**). Bone marrow aspiration demonstrated little fragmented bone tissue and fibrillar exudate, with IHC results showing CK-pan (+) and EMA (+), indicating metastatic carcinoma adjacent to the bone trabeculae. Based on these findings, the patient was diagnosed with poorly differentiated gastric adenocarcinoma (cT3N+M1, stage IVB).

**Table 1:** Laboratory data at the patient’s first visit (2023-12-18).

Blood components	Patient	Unit	Normal range
Complete blood count			
White blood cell	6.85	E+9/L	3.5-9.5
Red blood cell	1.69	E+9/L	3.8-5.1
Hemoglobin	56	g/L	115-150
Hematocrit	17.8	%	35-45
Mean corpuscular volume	105.3	fL	82.0-100.0
Platelet	50	E+9/L	125-350
Neutrophil	78.7	%	40-75
Lymphocyte	15.5	%	20.0-50.0
Monocyte	4.4	%	3.0-10.0
Eosinophil	1.3	%	0.4-8.0
Basophil	0.1	%	0.0-1.0
Coagulation test			
Activated partial thromboplastin time	37.3	s	28.00-43.00
Prothrombin time	15.5	s	11.00-15.00
Fibrinogen	1.38	g/L	2.0-4.0
D-dimer	>20	mg/L	0.0-0.5
Biochemistry			
Total protein	63.4	g/L	65.0-85.0
Albumin	40.6	g/L	40.0-55.0
Alanine aminotransferase	37	U/L	7-40
Alkaline hosphatase	459	U/L	35-135
Total bilirubin	24.9	μmol/L	0.0-21.0
Lactate dehydrogenase	269	IU/L	124-222
Biochemistry			
Blood urea nitrogen	5.69	mmol/L	2.60-8.80
Creatinine	52.00	μmol/L	41-81
Na (sodium)	142	mmol/L	137-147
K (potassium)	3.14	mmol/L	3.50-5.30
Cl (chlorine)	105.1	mmol/L	99.0-110.0
Glucose	5.23	mmol/L	3.9-6.1
Carcinoembryonic antigen	5.5	ng/mL	0.0-5.0
Carbohydr ateantigen 19-9	917.4	U/mL	0.0-43.0

**Figure 3:**
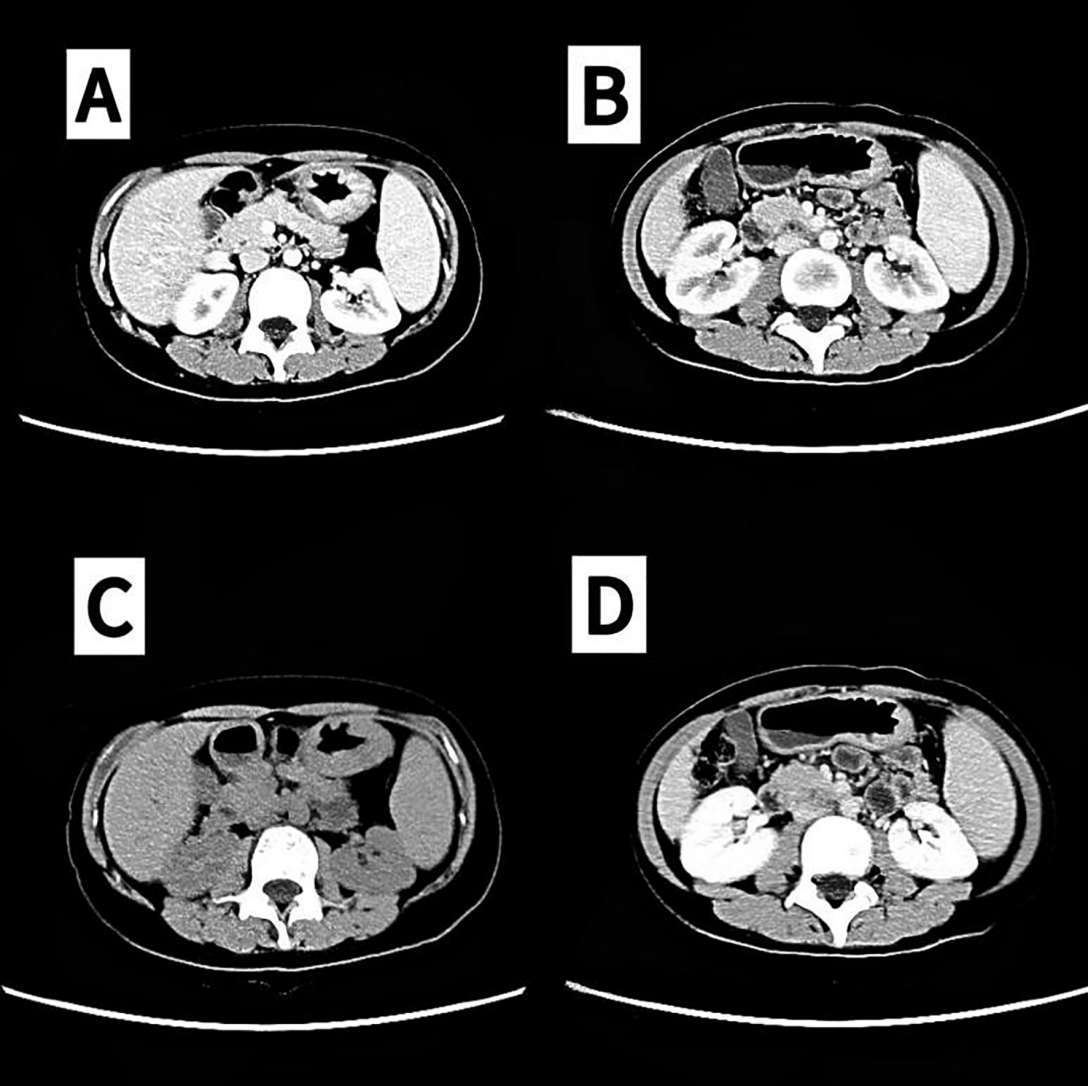
Partial remission (PR) confirmed by computed tomography (CT). **(A, C)** 2024-01-25. **(B, D)** In 2024-03-28, partial remission (PR) was assessed via CT (lymph nodes in the hepatogastric hiatus, splenogastric hiatus, and mesenteric root section were reduced and minimized from previous levels).

Treatment began on December 21, 2023, considering the patient’s DIC status and ECOG performance status of 2. Cardiac ultrasound performed on December 19, 2023, showed no significant abnormalities, with an ejection fraction (EF) of 66%. Chemotherapy was initiated with Docetaxel at a dose of 25 mg/m², which was increased to 30 mg. Fluorouracil was administered at a dose of 1300 mg/m², increased to 1.5 g, via intravenous pump for 24 hours. Trastuzumab was administered at a dose of 8 mg/kg (360 mg). On December 28, 2023, Docetaxel was continued at a total dose of 30 mg, and Fluorouracil was maintained at 1.5 g via intravenous infusion. During this period, platelet and red blood cell transfusions and cryoprecipitation therapy were provided. The patient also received 40 mg of OxyContin every 12 hours for pain management, resulting in a reduction of the NRS score to 0. Prophylactic G-CSF was administered 24 hours after chemotherapy. Deslizumab treatment was given in one 28-day cycle. The patient’s DIC resolved by December 27, 2023 (**Table 2**). In addition, the patient’s cancer pain was alleviated, leading to a gradual tapering off of OxyContin, which was eventually discontinued. On January 11, 2024, the chemotherapy regimen was switched to the standard-dose FLOT regimen, administered biweekly for 2 cycles, with another dose on January 25, 2024. Simultaneously, treatment was modified to include trastuzumab combined with cadonilimab every 3 weeks, continuing for 2 cycles on January 11, 2024, and March 1, 2024. Imaging assessment on January 25, 2024, showed a partial response (PR) (**Figure 3**). The patient then received 5 additional cycles of the FLOT regimen on February 8, February 22, March 8, March 22, and April 4, 2024, along with 2 cycles of trastuzumab plus cadonilimab therapy during this period. A grade II hypersensitivity reaction to oxaliplatin occurred during the final chemotherapy cycle. Imaging on March 27, 2024, indicated PR (**Figure 3**). On April 21, 2024, the treatment regimen was changed to S-1 (tegafur-gimeracil-oteracil) plus cadonilimab and trastuzumab as maintenance therapy. Follow-up imaging on November 28, 2024, revealed progressive disease (PD), prompting a switch to trastuzumab deruxtecan on November 29, 2024, which the patient continues to receive.

**Table 2:** DIC Laboratory Parameters Before and After Treatment (2023.12.08 vs 2023.12.27).

Blood components	Laboratory data		Unit	Normal range
	2023/12/08	2023/12/27		
Platelet Count	50.00	216.00	E+9/L	125-350
Prothrombin Time [PT]	15.50	1.02	s	11.00-15.00
Activated Partial Thromboplastin Time [APTT]	37.30	34.00	s	28.00 - 43.00
Fibrinogen [FIB]	1.38	4.32	g/L	2.00 - 4.00
Thrombin Clotting Time [TT]	19.00	14.70	s	14.00 - 21.00
Prothrombin Time Activity [PT%]	74.00	96.00	%	80.00 - 120.00
D - Dimer [DD]	>20	9.07	mg/L FEU	0 - 0.50

## 3 Discuss

GC patients have a low incidence of bone metastasis, but the prognosis remains poor. A large-scale study of 4,617 GC cases reported a bone metastasis rate of 3.8%, with OS ranging from 4 to 5 months (11). Notably, GC patients who present with bone pain as the initial symptom, without preceding gastrointestinal manifestations, are often misdiagnosed with orthopedic conditions, leading to diagnostic delays (12). In this case, the female patient initially presented with bone pain and was subsequently diagnosed with metastatic GC after comprehensive evaluations, including PET/CT, gastroscopy, and pathological analysis. This highlights the importance of a thorough diagnostic workup, with PET/CT playing a critical role in early detection of the primary tumor. Earlier studies have demonstrated that patients with GC and bone metastasis are prone to bone marrow invasion, and compared to conventional metastatic gastric cancer, BMM gastric cancer exhibits significant differences in clinical pathology and molecular mechanisms. These patients more frequently exhibit hematologic abnormalities such as thrombocytopenia, elevated alkaline phosphatase (ALP), anemia, and DIC, reflecting bone marrow involvement and a pro-thrombotic tumor microenvironment (13). In addition, histologically, BMM is associated with diffuse-type tumors and signet ring cells, and biologically, the bone marrow niche provides a highly vascularized and immunosuppressive environment that facilitates tumor colonization (14). Besides, research has revealed that cytokines and growth factors such as PDGF, VEGF, and TGF-β promote osteoblastic reactions and further enhance tumor progression in the bone microenvironment (15). Therefore, these biological differences justify treatment strategies that aim not only to control systemic tumor burden, but also to stabilize coagulation and bone remodeling processes. Tragically, the patient’s initial diagnosis was GC with DIC and BMM, as suggested by the bone marrow biopsy results. A single-cell study suggests that the core mechanism of highly aggressive gastric cancer-associated DIC involves NETosis driven by immature neutrophil expansion, synergistic angiogenic signaling and myeloid immunosuppression, leading to microthrombosis (16). Prior research report a 3.74% incidence of DIC in GC patients, with a median OS of only 2.867 months, potentially linked to tumor-derived procoagulant substances or concurrent infections (6, 17). Moreover, malignant cell infiltration into the bone marrow in GC patients with BMM can lead to DIC, anemia, thrombocytopenia, and other hematological disorders. Further research has identified gastrointestinal bleeding, multiple metastatic patterns, and poorly differentiated histopathology as significant risk factors for DIC in GC, with these cases showing a median survival of just 57 days (18). However, our patient is CPS PD-L1-negative and MSS, which may offer some insights into treatment options. Remarkably, despite these challenges, our patient achieved an OS of 15 months through aggressive antitumor therapy combined with comprehensive supportive care. This case emphasizes the critical importance of active antitumor treatment in GC patients with BMM complicated by DIC, while also highlighting the vital role of meticulous supportive care in improving clinical outcomes.

Histopathological examination confirmed poorly differentiated gastric adenocarcinoma. IHC analysis demonstrated HER-2 expression (2+), while FISH yielded negative results. Intriguingly, plasma NGS revealed a markedly elevated ERBB2 copy number (34.88), creating a diagnostic discordance between tissue-based and liquid biopsy findings. We hypothesize that this discrepancy likely reflects significant intratumoral heterogeneity. The endoscopic biopsy specimen, obtained from a single gastric lesion, may not have captured the complete molecular profile of the tumor. In contrast, plasma NGS analyzed circulating tumor DNA (ctDNA) derived from multiple metastatic sites, including bone marrow lesions, potentially providing a more comprehensive representation of the tumor’s molecular characteristics. This phenomenon underscores both the limitations of single-site tissue sampling and the complementary value of liquid biopsy in metastatic gastric cancer. Previous studies have shown that approximately 30% of gastric cancers exhibit HER-2 heterogeneity (19). This heterogeneity may cause some patients to miss the opportunity for targeted therapy. Furthermore, existing literature comparing IHC and NGS for HER-2 detection in gastric cancer have shown that 16.5% of patients were HER-2 3+ by IHC, while NGS identified HER-2 mutations in 4.1% and copy number increases in 14.9% of patients. The concordance rate between IHC and NGS was 80%, and agreement between the two methods was 88.9% (19). Moreover, another study demonstrated 98.4% overall concordance (248/252) between NGS-detected HER-2 amplification and IHC/FISH results in breast and gastroesophageal adenocarcinomas, with discrepancies mostly occurring in cases of low tumor content or HER-2 heterogeneity (20). Unfortunately, in this case, the patient had limited tissue obtained from the initial endoscopic biopsy, leaving no remaining pathological material for HER-2 reevaluation. The JACOB trial indicated that HER-2 copy-number variation (CNV) assessed by NGS could correlate with better objective response rate (ORR), (PFS, and OS (21). Therefore, NGS offers a more sensitive method for identifying HER-2 positive patients, particularly when tissue samples are limited. Moreover, HER-2 copy number levels may serve as a predictor for response to anti-HER-2 therapy. In this case, despite the small gastric biopsy sample showing HER-2 (2+) and negative FISH, plasma NGS revealed a high HER-2 copy number, which likely reflects tumor heterogeneity. Consequently, trastuzumab combined with chemotherapy was chosen for treatment. The ToGA study established the efficacy of trastuzumab in metastatic gastric cancer treatment. However, the question remains: can adding ICIs to trastuzumab and chemotherapy further improve outcomes for HER-2 positive metastatic GC? The KEYNOTE-811 trial demonstrated that pembrolizumab combined with trastuzumab and chemotherapy significantly improved PFS in HER-2 positive metastatic gastric/gastroesophageal junction adenocarcinoma patients with PD-L1 CPS ≥ 1. However, grade ≥3 treatment-related adverse events occurred in 58% of patients versus 51% in the placebo group (10). Another Phase II trial showed that FOLFOX plus nivolumab and trastuzumab achieved a 12-month OS of 70% in previously untreated HER-2 positive metastatic gastroesophageal adenocarcinoma, superior to historical ToGA data. PD-L1 CPS expression did not predict prognosis, and 82% of patients experienced grade ≥3 adverse events (commonly leukopenia, infections, fatigue, and neurotoxicity) (22). Thus, ICIs combined with trastuzumab and chemotherapy have been shown to be both effective and safe for HER-2 positive metastatic GC. This patient also received this combination regimen, achieving improved outcomes with well-managed adverse effects.

Although several clinical studies have confirmed that ICIs combined with chemotherapy can improve the prognosis of patients with HER-2-negative metastatic gastric cancer (GC) (23), the benefit is predominantly observed in patients with positive CPS PD-L1 expression. A meta-analysis confirmed that the clinical benefit of ICIs in gastroesophageal adenocarcinoma patients is correlated with CPS PD-L1 expression, with higher CPS PD-L1 levels leading to more pronounced improvements in OS. In contrast, patients with CPS PD-L1 <1 did not show an improvement in OS. Furthermore, a PD-L1 CPS ≥5 threshold has been suggested as a way to optimize the risk/benefit ratio of ICIs (24). Our patient had a CPS PD-L1 <1 and, therefore, might not have benefited from treatment with ICIs alone. The COMPASSION-15 study evaluated cadonilimab, the world’s first PD-1/CTLA-4 bispecific antibody, in combination with chemotherapy as a first-line treatment for HER-2-negative metastatic adenocarcinoma of the stomach or gastroesophageal junction. The study found that the median OS in the cadonilimab group was significantly prolonged compared to the placebo group [median OS 15.0 months vs. 10.8 months, HR 0.62 (95% CI: 0.50–0.78), P<0.001]. In the PD-L1 CPS ≥5 population, the median OS was not yet reached in the cadonilimab group versus 10.6 months in the placebo group (HR 0.56, P<0.001). In the PD-L1 CPS <5 population, the median OS was 14.8 months in the cadonilimab group compared to 11.1 months in the placebo group (HR 0.70, P=0.011). The cadonilimab group experienced a 65.9% incidence of grade 3 treatment-related adverse events, primarily hematologic toxicity, with most immune-related adverse events being grade 1 or 2, compared to 53.6% in the placebo group (25). Additionally, a case report described a patient with HER-2-positive metastatic gastroesophageal junction adenocarcinoma treated with cadonilimab in combination with chemotherapy, resulting in complete remission (CR) (9). In a case report, a 43-year-old male diagnosed with diffuse poorly differentiated gastric adenocarcinoma indicated HER-2 positivity by IHC, a PD-L1 CPS of 1, and microsatellite instability. After treatment with XELOX combined with trastuzumab and sintilimab, a 2-month follow-up assessment revealed PR, with a PFS of 10 months (7). Given the patient’s high NGS HER-2 CN expression, PD-L1 CPS < 1, and MSS status, we opted for a treatment regimen combining cadonilimab, trastuzumab, and chemotherapy for this GC patient. The patient remains alive with good quality of life.

The prognosis of patients with GC undergoing BMM and concurrent DIC is poor due to acute onset, poor physical condition, and hematological abnormalities, which complicate the assessment of chemotherapy tolerance, often leaving physicians in a therapeutic dilemma. Studies have shown that the combination of docetaxel and fluorouracil can effectively reduce platelet consumption, and patients with DIC-positive GC and BMM respond more quickly to chemotherapy, with a median DIC resolution time of 12 days (range 4–19 days) and a median DIC-free time of 137 days (range 6–457 days). The median OS for patients with DIC resolution was 7.200 months, compared to only 2.067 months for those who did not receive chemotherapy. This confirms that chemotherapy using docetaxel combined with fluorouracil can rapidly alleviate DIC in patients with gastric cancer complicated by DIC, thereby improving their prognosis (6). Case reports indicate that GC with BMM is prone to DIC, and maintenance therapy with TS-1 following initial treatment with the SOX regimen can achieve a PFS of over a year in GC patients with BMM and concurrent DIC (26). Therefore, under adequate supportive care, patients with GC, BMM, and DIC can benefit from chemotherapy. However, the role of ICIs in this patient population remains unproven by clinical studies, though case reports suggest that a treatment strategy combining PD-1 inhibitors with chemotherapy can improve survival and coagulation function in GC patients with BMM and DIC, with good safety profiles (7). Additionally, case reports have shown that trastuzumab combined with TS-1 can also improve survival in GC patients with BMM and DIC (27). Moreover, the clinical trial have shown that FLOT regimen in treating metastatic adenocarcinoma of the stomach or gastroesophageal junction yields an ORR of 57.7% and DCR of 80.8%, with PFS and OS of 5.2 and 11.1 months, respectively (28). Taken together, the patient’s complex clinical presentation, encompassing BMM, an initial ECOG performance status of 2, and active DIC, necessitated a carefully staged therapeutic approach. Following multidisciplinary team (MDT) consensus, we implemented a stepwise strategy. In the course of initial phase (DIC Control), a modified low-dose intensive regimen (weekly docetaxel 25 mg/m² + fluorouracil 1300 mg/m²) was initiated to achieve rapid hematologic stabilization and symptomatic relief. This approach successfully resolved DIC within two weeks while significantly alleviating cancer-related pain. Subsequently, under intensification phase (Disease Control), upon achieving hematologic stability and performance status improvement, and therapy was escalated to standard-dose FLOT combined with cadonilimab and trastuzumab. This combination aimed to maximize tumor burden reduction while capitalizing on potential synergistic effects between HER2-targeted therapy and immune checkpoint inhibition. Ultimately, For the duration of maintenance phase (Long-term Management), following six cycles of intensive therapy and confirmed PR, the regimen was transitioned to S-1 with cadonilimab and trastuzumab. This maintenance protocol balanced sustained disease control with reduced treatment-related toxicity, enabling prolonged therapy duration while preserving quality of life. Furthermore, trastuzumab deruxtecan (T-Dxd), an ADC drug targeting HER-2 targets. A single-arm phase II DESTINY-Gastric02 study was given single-agent T-Dxd treatment for patients with HER2-positive advanced gastric and gastroesophageal junction adenocarcinoma who had progressed or intolerable after first-line treatment with trastuzumab. The main study endpoint was objective response rate (ORR). The results showed that T-Dxd had a good ORR (41.8%) in second-line treatment, with a median PFS of 5.6 months and a median OS of 12.1 months, which was better than the ORR and median PFS data of single-agent chemotherapy in prior research (29). While HER2-targeted-ADCs show promising potential to overcome tumor heterogeneity in HER2-positive metastatic GC, their efficacy as first-line therapy requires further clinical validation. In this context, considering both the ongoing investigation of ADCs in first-line settings and the patient’s indolent disease progression, we implemented a sequential treatment strategy: initial trastuzumab-based therapy followed by ADCs upon progression. Subsequent transition to T-DXd after disease progression yielded exceptional outcomes, with PFS reaching 12 months and OS extending to 15 months. The treatment demonstrated a favorable safety profile, with hematologic toxicity (predominantly grade 1-2) being the most common yet manageable adverse effect. This approach not only achieved significant survival benefit but also maintained excellent functional status, enabling preserved performance of daily activities (ECOG 0).

In summary, patients with GC and BMM are at high risk of developing DIC. Although clinical management poses significant challenges, aggressive antitumor therapy can improve outcomes in these patients. This case demonstrates that cadonilimab combined with trastuzumab and chemotherapy may improve prognosis in HER-2-positive metastatic GC patients with BMM and DIC, with a manageable safety profile. However, further clinical studies are warranted to validate these findings.

## Data Availability

The original contributions presented in the study are included in the article/supplementary material. Further inquiries can be directed to the corresponding author/s.
